# Geographical Characteristics of *Culex tritaeniorhynchus* and *Culex orientalis* Microbiomes in Korea

**DOI:** 10.3390/insects15030201

**Published:** 2024-03-18

**Authors:** Jeong-Hyeon Lee, Hee-Il Lee, Hyung-Wook Kwon

**Affiliations:** 1 Department of Life Sciences, Incheon National University, Yeonsu-gu, Incheon 22012, Republic of Korea; jeonghyeon@inu.ac.kr; 2 Convergence Research Center for Insect Vectors, Incheon National University, Yeonsu-gu, Incheon 22012, Republic of Korea; 3 Division of Vectors and Parasitic Diseases, Korea Disease Control and Prevention Agency, Heungdeok-gu, Cheongju 28159, Republic of Korea

**Keywords:** mosquito, *Culex tritaeniorhynchus*, *Culex orientalis*, microbiome, biomarker

## Abstract

**Simple Summary:**

Mosquitoes, vectors of diseases like Japanese encephalitis, harbor complex microbial communities that influence their biology and disease transmission. The microbiome of *Culex tritaeniorhynchus* and *Culex orientalis*, key vectors of the Japanese encephalitis virus, remains poorly understood. Utilizing 16S rRNA Illumina sequencing, we investigated the microbiomes of these mosquitoes collected across diverse regions in the Republic of Korea. Our analysis revealed the presence of 36 prevalent bacterial families, with microbial composition variations predominantly influenced by geographic location. Moreover, the identification of species-specific biomarkers underscores the potential for ecological niche differentiation between *Culex tritaeniorhynchus* and *Culex orientalis*. This research sheds light on the intricate relationship between mosquito vectors, their microbiomes, and environmental factors, offering insights into vector ecology and disease transmission dynamics.

**Abstract:**

Mosquitoes, the primary vectors of arboviruses, harbor a diverse microbiome that plays a crucial role in their development, immunity, and vector competence. The composition of the mosquito microbiome is heavily influenced by the environment and habitats. Therefore, identifying the relationship between the habitat and the mosquito’s microbial community can improve the overall understanding of mosquito biology. However, The microbiome profiles of *Culex tritaeniorhynchus* and *Culex orientalis*, known as transmission vectors of the Japanese encephalitis virus, are poorly understood. Using 16S rRNA Illumina sequencing, we hereby investigated the microbial profiles in these two mosquito species collected in several areas in the Republic of Korea. Thirty-six prevalent bacterial families were identified from these mosquito species. The microbial composition variations were primarily influenced by the mosquito collecting sites. Moreover, species biomarkers were identified by utilizing the regional specificity of the mosquito microbiome. Based on the microbiome profiles representing high similarity, *Culex orientalis* may share an ecological niche with *Culex tritaeniorhynchus*.

## 1. Introduction

Mosquitoes constantly interact with microorganisms throughout their lives [1]. Mosquitoes interact with diverse plant nectar in their habitat, and blood-feeding from mammalian animals is essential for their egg reproduction [2]. In the course of these activities, mosquitoes acquire numerous bacteria [3,4]. The mosquito microbiome consists of the core microbiome, which is essential for basic survival, and the environmental microbiome, which can only be obtained in specific environments. The core microbiome is typically defined as the assemblage of members shared among microbial communities from similar habitats [5]. The regional specificity of organisms is largely dependent on their core microbiome and can be utilized for tracing mosquito habitats [6]. Indeed, differences were observed in the results of mosquito microbiome analysis across various countries and regions [7,8,9,10]. Moreover, these microorganisms have demonstrated their potential to mitigate disease transmission by decreasing mosquito lifespan and impeding the establishment of pathogens or parasites through natural competitive interactions [4,11,12]. Recent studies have illuminated the involvement of microbiota, including *Wolbachia*, *Serratia*, *Asaia*, *Pantoea*, and *Chromobacterium*, in disease transmission. This is particularly evident in mosquito-borne diseases such as Zika, dengue fever, chikungunya, and malaria [12,13,14,15,16,17,18]. 

*Wolbachia*, a common endosymbiotic bacterium found in insects, including mosquitoes, has been extensively studied for its potential to control mosquito-borne diseases [12]. Its presence in mosquitoes can disrupt the replication and transmission of pathogens such as dengue, Zika, and malaria parasites while also inducing reproductive abnormalities that result in reduced mosquito lifespan and population suppression [11,13]. Indeed, introducing the wMel strain of *Wolbachia pipientis* into the *Aedes aegypti* population proved beneficial in lowering the prevalence of clinical dengue as well as the number of dengue fever hospitalizations [14]. These microorganisms represent promising candidates for reducing mosquito lifespan and impeding the establishment of pathogens or parasite microbiomes through natural competitive interactions [4]. The primary vector of JEV is *Culex tritaeniorhynchus*, which was prevalent in countries in Africa, the Middle East, southern Europe, and Asia [19]. Moreover, 13 species of mosquitoes, including *Cx. quinquefasciatus* and *Cx. pipiens*, the secondary vectors carrying JEV, were identified and reported consistently. Recently, JEV was also confirmed from *Cx. orientalis* in Korea [20]. 

While the impact of the mosquito microbiome on pathogen transmission has been well recognized, the microbiome profiles of JEV vectors in ROK remain largely unexplored. Understanding the microbiome composition of existing mosquitoes is crucial for the successful implementation of microbial-based mosquito control strategies. This study, therefore, aimed to investigate the microbiome profile of *Cx. tritaeniorhynchus*, known as JEV vector in ROK. Moreover, region-specific microbiomes were identified, affirming the potential for tracking mosquito habitats. Furthermore, the microbiome of *Cx. orientalis*, a species in which the JEV virus was recently detected, was also analyzed. 

## 2. Methods

### 2.1. Mosquito Collection and DNA Extraction

Adult females *Cx. tritaeniorhynchus* and *Cx. orientalis* were collected from 12 rural locations in the Republic of Korea during 1–15 August 2021 with the support of the Regional Center for Vector Surveillance against Climate Change supported by the Korean Disease Control and Prevention Agency [21]. The sites are located at migratory bird habitats countrywide. Two CDC black-light traps (The John W. Hock Co., Gainesville, FL, USA) and one BG-Sentinel™ trap (Biogents, Regensburg, Germany) were utilized using dry ice used as bait. The collecting chamber of traps was cleaned with 70% ethanol before use. Following collection, all mosquitoes were transferred to our lab under ice packs in Styrofoam coolers. Female mosquitoes were morphologically separated and identified using an optical microscope and taxonomy keys by well-trained experts in this laboratory [22]. Other mosquitoes, such as *Cx. Pipiens* complex, *Aedes vexans*, and *Anopheles* spp. were also collected for DNA extraction, *Cx. tritaeniorhynchus* and *Cx. orientalis* were put in sterile, Dnase- and RNAse-free 1.5 mL tubes and stored at −80 °C until use.

To minimize bias derived from population variation, up to 10 mosquitoes were pooled in a group. For a site where fewer than 10 mosquitoes were collected, all mosquitoes were pooled for downstream analysis. The genomic DNA was extracted from all adult female mosquitoes under aseptic conditions using the Power Soil Kit (QIAGEN, Hilden, Germany) according to the manufacturer’s instructions. Following quality and quantity assays, DNA samples were used for 16s metagenomics analyses.

### 2.2. Sample Preparation, 16s Sequencing, and Taxonomic Analysis

DNA sample preparation, 16s sequencing, and taxonomic analyses were performed as described previously [23]. PCR amplification, sample processing, 16S rRNA gene sequencing, and taxonomic analysis were performed by the National Instrumentation Center for Environmental Management (NICEM, www.nicem.snu.ac.kr, accessed on 1 October 2021), Republic of Korea, and performed commercially. The V3–V4 region of 16S rRNA was amplified using primers (Supplementary Table S1) and KAPA HiFi HotStart ReadyMix (Roche, cat# kk2601). PCR thermal cycle was: denaturation at 96 °C for 3 min, Amplification 30 cycles at 96 °C for 30 s, at 55 °C for 30 s, at 72 °C for 30 s, and final extension at 72 °C for 5 min. PCR amplicons were run on 1.2% agarose gels to check bands and intensity. The amplified DNAs were purified using Ampure XP beads (Beckman, Indianapolis, IN, USA). With reference to DNA bands and molecular weight, the amplicons were pooled into the same quantities and used to produce Illumina DNA libraries. The libraries were sequenced using Illumina MiSeq runs to collect 2 × 300 bp paired-end reads. Using the Chunlab analytical pipeline PKSSU 4.0 DB [24], all collected sequence data were evaluated. Short assemblies, as well as reads with ambiguous base calls, were excluded from the read collection. By grouping reads at 3% divergence or 97% similarity, reads were denoised and allocated to an amplicon sequence variant (ASV). Chimeric sequences not linked to an ASV were excluded. “Unclassified” ASVs were not taxonomically categorized and excluded from further analysis. For pooled samples, FASTAQ mapping files were created using NICEM at various taxonomic levels, and the reads were presented proportionally to the total reads associated with each ASV.

### 2.3. Data Analysis Using QIIME2 

DNA sequence data were analyzed using QIIME2, as described previously [23,25]. The reads were denoised and trimmed using the DADA2 pipeline [26]. The alpha and beta diversity indices were constructed using the QIIME2 diversity plugin with a sampling depth set at 788. The bacterial diversity of mosquitoes between species and collecting sites was compared using variety indices. Using QIIME2, Shannon and evenness diversity indices of mosquito samples were calculated from bacterial ASV count data. The Kruskal-Wallis H test was used to compare these results among species. The difference in mosquito profiles between species was evaluated using PERMANOVA. A significant difference in bacterial profiles was analyzed using pairwise PERMANOVA in QIIME2. Microbiomes abundant in different species were searched using the ANCOM plugin [27] in QIIME2. The effect size for differential abundance was set at log F20 and W20.

### 2.4. Bacterial Profiles

Mosquito samples from the sites where both species were collected were selected for further analysis. After annotation, bacterial reads constituting less than 1% were grouped as “<1%”. Samples with more than 30% of “<1%” were excluded. Samples Ct2, Ct7, and Co8 were excluded from the pair-wise analysis (Supplementary File S1). After quality verification, reads from locations 4, 5, 6, 9, 10, and 11 were selected for further analysis. At the phylum, family, and species levels, read count and abundance data were evaluated for bacterial ASVs. The percentage abundance of bacterial ASV was calculated for all samples. The average and maximum abundance of bacterial families in the dataset were used to identify abundant bacterial families. At the species level, unclassified ASVs were representative at the highest categorization level feasible. 

### 2.5. Taxonomic Biomarker

Taxonomic biomarkers were searched as previously reported [23]. The abundance of data from each mosquito species was analyzed for the presence/absence of microbes. When a species was found in one sample, it was considered present. The average microbiome profile of all sites was compared, and taxonomic biomarkers were extracted using a plugin Comparative Analyzer for MTP sets of Ezbiocloud. Taxonomic biomarkers were considered significant when the *p*-value was less than 0.05. The top three taxa showing the highest abundance were chosen as taxonomic biomarkers of the site, with the exclusion of an abundance ratio of less than 1%.

### 2.6. Statistical Analysis

Wilcoxon rank-sum test, PERMANOVA (Permutational multivariate analysis of variance), Kruskal-Wallis H test, and LEfSe (Linear discriminant analysis Effect Size) were performed using R version 4.1.0 in RStudio Version 1.4.1106 [28] and EZbiocloud [24].

## 3. Results

### 3.1. Collected Mosquitoes and DNA Reads

**Figure 1:**
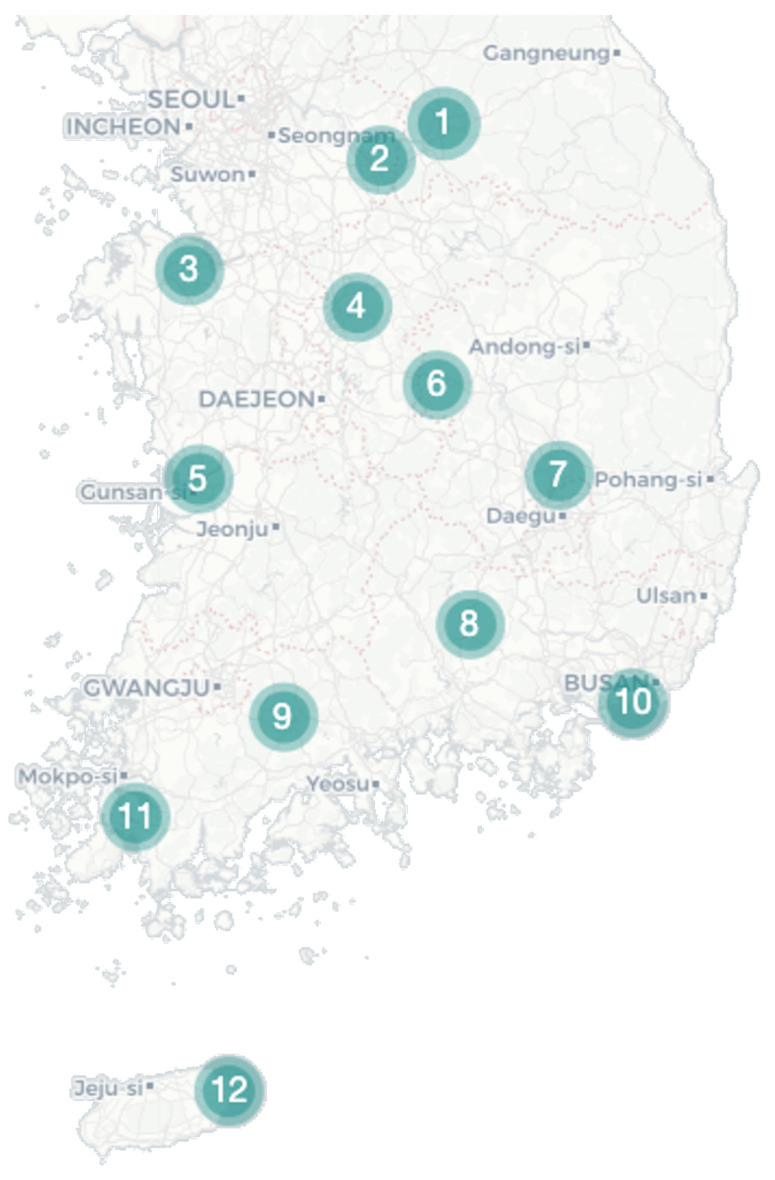
Collecting site. Mosquito samples were collected using Blacklight (BL) and Biogent (BG) traps from 12 rural areas in the Republic of Korea to establish the regional microbiome profile of *Culex tritaeniorhynchus* and *Culex orientalis*.

During August 2021, *Cx. tritaeniorhynchus* and *Cx. orientalis* were collected at 12 bird sanctuaries across the country, with both species being found at nine sites (Figure 1). *Cx. tritaeniorhynchus* was predominantly located in the southern region, with the highest collection at Site 8, followed by Sites 11 and 9 (Table 1). In contrast, *Cx. orientalis* was primarily identified in the northern region, with the highest abundance recorded at Site 4, followed by Site 7 (Table 1). After denoising the collected reads, a total of 536,574 valid reads remained (Supplementary File S1). The sufficiency of the selected valid reads for further analysis was indicated in a rarefaction graph (Supplementary Figure S1). 

### 3.2. Alpha and Beta Diversities by Mosquito Species

**Figure 2:**
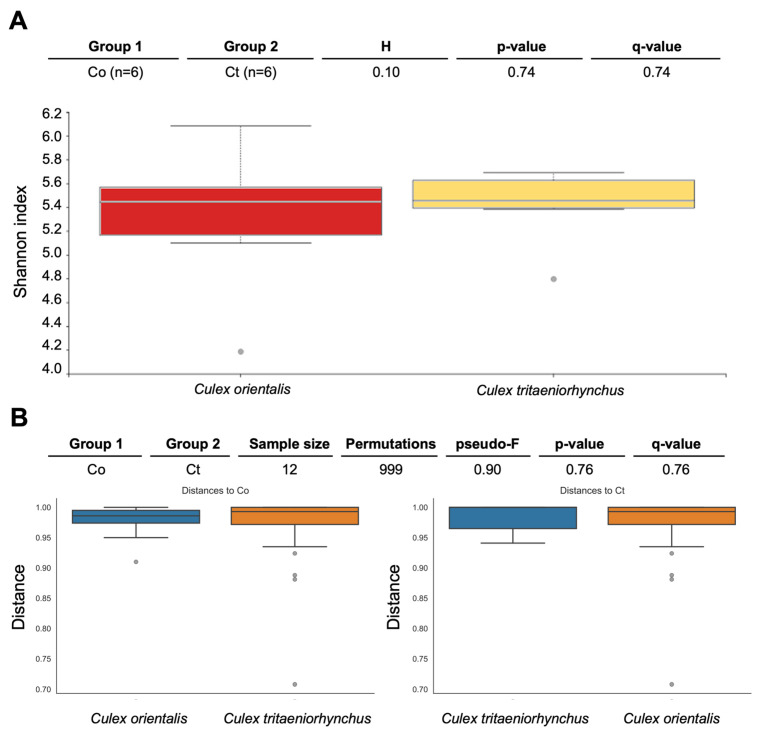
Pairwise diversity comparisons of the microbiome of *Culex tritaeniorhynchus* and *Culex orientalis*. Each box represents a distinct bacterial diversity metric for mosquito samples, which are grouped by mosquito species. The Shannon in each mosquito sample is depicted in panel (**A**), with no significant difference between groups (Kruskal-Wallis: H = 0.10, *p* = 0.74). (**B**) PERMANOVA (999 permutations) tests with Benjamini-Hochberg FDR correction were used to make these comparisons (q-value). Two species of mosquitoes collected at Sites 4, 5, 6, 9, 10, and 11 were used for analysis.

**Figure 3:**
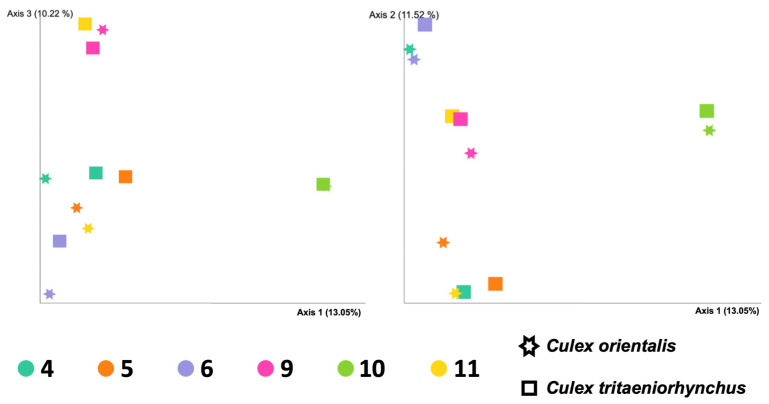
Principal coordinated analysis (PCoA) plots of the Bray-Curtis distances of adult female *Culex tritaeniorhynchus* and *Culex orientalis* microbiome diversity according to collecting sites.

Site 9 showed the highest Shannon index for *Cx. orientalis*, indicating significant microbial diversity. A significant regional difference was observed in *Cx. orientalis*. In contrast, *Culex tritaeniorhynchus* exhibited the highest Shannon index at Site 6, but no significant difference was observed across sites. Alpha diversity group significance analysis revealed no significant difference between *Cx. orientalis* and *Cx. tritaeniorhynchus* (Figure 2A). Similarly, the PERMANOVA analysis for both species did not yield significant values (Figure 2B). The PCoA graph visually represented the distance between each sample based on microbiome composition and the ratio of all samples (Figure 3). Notably, clusters corresponding to sites were formed by Sites 5, 6, 9, and 10. Interestingly, mosquitoes of the two species at Sites 4 and 10 were considerably distant from mosquitoes in other sites.

### 3.3. Microbiome Taxonomy by Samples

**Figure 4:**
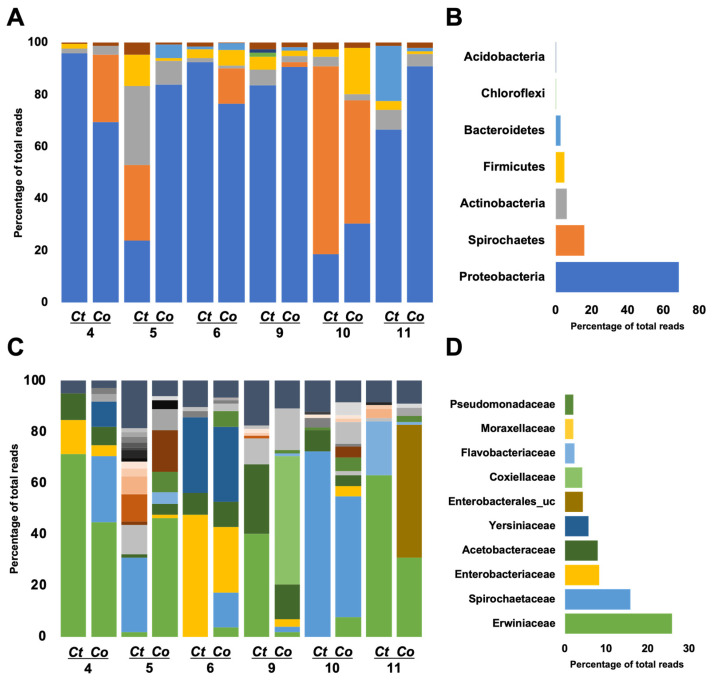
Microbiome profiles are conducted at the family level of *Culex tritaeniorhynchus* and *Culex orientalis*. Each bar reflects a microbiome profile, which is colored based on the family level allocated to a phylum. Left side-bar plots represent the microbiome profiles of adult female *Cx. tritaeniorhynchus* and *Cx. orientalis* mosquitoes collected from 6 sites (**A**: phylum level, **C**: family level). Each bar represents the microbiome profile of the pooled sample by species (Ct: *Cx. tritaeniorhynchus*, Co: *Cx. orientalis*). Right side-bar plots represent the average of all regions (**B**: phylum level, **D**: family level).

The microbiome of the two mosquito species was categorized into seven phyla, with Proteobacteria, Spirochaetes, Actinobacteria, and Firmicutes collectively representing over 95% of the total composition (Figure 4A). Proteobacteria exhibited dominance across the sites (Figure 4B). Specifically, in *Cx. tritaeniorhynchus* (Ct) 4 and 6, as well as *Cx. orientalis* (Co) 11 and 9, Proteobacteria constituted over 90%, while in Ct 5 and 10 and Co 10, it was less than 30%. Site 10 displayed dominance of Spirochaetes, exceeding 50% for both Co and Ct. Firmicutes reached the highest proportion in Co 10. At Site 5, the prevalence of Actinobacteria was notably higher compared to other sites, particularly in *Cx. tritaeniorhynchus*.

**Figure 5:**
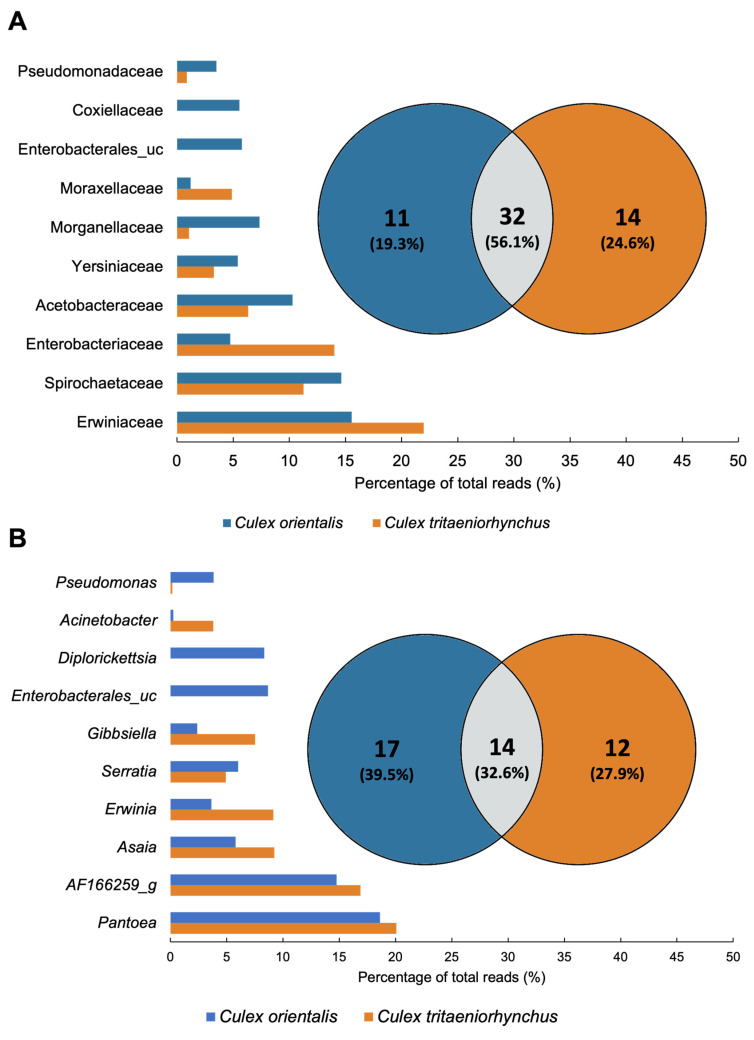
Comparison of *Culex tritaeniorhynchus* and *Culex orientalis* microbiome profile. Venn diagrams were prepared for the presence/absence data of mosquito samples at (**A**) Family and (**B**) Genus levels. The microbiome unique to samples or shared in common by distinct groups is shown by numbers. The proportion of total reads of the microbiome is represented by bar plots.

A total of 32 families were classified, with the top 10 families, based on the highest average ratio, collectively contributing to over 70% of the total abundance (Figure 4C,D and Figure 5A). The predominant family was Erwiniaceae, particularly dominant in Ct 4, 9, 11, and Co 4 and 5. Erwiniaceae was present in 10 samples but absent from Ct 10 and 6. Spirochaetaceae was the next most abundant family, prevalent in six samples, with the highest occurrence at Ct 10, Co 10, and dominance at Ct 5. Enterobacteriaceae and Yersiniaceae exhibited high detection rates in Ct 6 and Co 6, with Yersiniaceae additionally detected in Ct 6, Co 4, 5, and 6. Acetobacteraceae showed high prevalence in both Co and Ct at Site 9. Co 9 was uniquely dominated by Coxiellaceae, and Co 11 exhibited dominance by Enterobacterales_uc. 

### 3.4. Difference of Microbiome between Species and Sites

A total of 43 genera were detected from the two mosquito species. In *Cx. tritaeniorhynchus*, 26 genera were identified, while *Cx. orientalis* exhibited 31 genera, with 14 genera shared between the two mosquito species (Figure 5B). Pantoea, AF166259_g, Asaia, Erwinia, and Serratia were shared genera that dominated in both mosquito species. Pantoea was present in all samples except Ct 6, 9, and 10. It was notably abundant in Ct 4, 11, and Co 4, 5, and 11. AF166259_g was detected at a high rate at Site 10. Asaia was identified in all samples except Site 11, and its prevalence was particularly notable at Site 9. Enterobacterales_uc and Diplorickettsia were exclusive to *Cx. orientalis*. Microbiomes specifically identified in the collection site, serving as potential regional biomarkers, were selected from Sites 4, 5, 6, and 10. The identified regional markers encompass the Pantoea JTJJ_s group at Site 4, Micrococcales at Site 6, Gibbsiella quercinecans at Site 6, and AF166259_g at Site 10 (Table 2).

## 4. Discussion

The microbial community composition of mosquitoes varies depending on their habitats [18,29,30,31,32,33]. These microbiome characteristics have been utilized to establish the microbial community profile of mosquitoes across diverse regions [34]. In this study, we identified regional variations in the microbiome of two mosquito species inhabiting the ROK, and species biomarkers were identified to trace their habitats. The mosquito microbiome comprises a core microbiome essential for basic physiological functions and an environmental microbiome specific to particular habitats [35]. These characteristics of the mosquito microbiome were utilized to identify species biomarkers by considering the regional specificity and abundance of microorganisms [23]. These species biomarkers will be used as crucial biological clues for tracing mosquito movement. In this study, species biomarkers were identified from four sites, indicating the potential for tracking the habitats of mosquitoes in Korea. The coastal proximity of Sites 5, 10, and 11 is a noteworthy consideration. Given Korea’s peninsula nature, with three sides exposed to the sea, regional ecosystems could differ. Indeed, Sites 5 and 11 exhibited significantly different microbiome patterns from other samples, starting at the phylum level (Figure 4). The mosquito microbiome is influenced by diverse environmental factors, including the aquatic habitat of larvae and the diet or bloodsucking behavior of adults. Future research should focus on establishing a comprehensive database by concurrently gathering data on both mosquitoes and their surrounding environment. The predominant microbiome in *Cx. tritaeniorhynchus* in Sri Lanka comprised Erwiniaceae, Staphylococcaceae, and Bacillaceae [36], which aligns with our current study. However, Bacillaceae was absent in domestic mosquitoes. In comparison to other studies, our findings diverge particularly in the dominance of specific microbial taxa. Unlike studies on *Cx. pipiens* complex and *Cx. quinquefasciatus*, where *Wolbachia* was prevalent, our investigation revealed a predominance of microbiomes such as Erwiniaceae and Staphylococcaceae [37,38]. This discrepancy emphasizes the variability in microbial composition across different mosquito species and geographical regions. The establishment of species-specific regional biomarkers through microbiome analysis on potentially importable mosquitoes could enable the monitoring of imported mosquitoes. Creating a thorough microbiome database for mosquitoes in the Republic of Korea through spatiotemporal surveys is crucial for enhancing vector surveillance.

Our findings revealed a high similarity in the microbiomes of two distinct mosquito species. The two species shared 14 microbial communities (32.6%) at the genus level, without significant differences observed in diversity and differential abundance, indicating similar ecological niches when cohabiting in the same environment. However, at Site 5, despite being collected from the same area, the microbiome composition of the two species exhibited significant differences. Microbial community composition is influenced by the host’s genetic background, prolonged interactions with bacteria, and relationships between the microbiome and infectious pathogens. Previous studies have demonstrated distinct profiles in mosquito microbiomes in areas with and without a high prevalence of malaria [23]. Given the past detection of JEV in *Cx. orientalis*, there is a need for intensive research on the potential transmission of JEV by this mosquito species [20].

In this study, the co-occurrence of *Pantoea agglomerans* and *Asaia bogorensis* on both *Cx. tritaeniorhynchus* and *Cx. orientalis* is noteworthy. *Pantoea agglomerans* exhibited dominance in the midgut of *Anopheles funestus* and *Anopheles gambiae*, which were collected from Kenya and Mali [39,40]. This bacterium has also been identified in *Aedes albopictus* in North America [41]. *Pantoea agglomerans* have been documented as a microorganism capable of producing and secreting anti-Plasmodium effector proteins in the midgut of anopheline mosquitoes [42]. There is a need to investigate whether *Pantoea* contributes to protection against the JEV in *Cx. tritaeniorhynchus* and *Cx. orientalis*. Similarly, both *Cx. tritaeniorhynchus* and *Cx. orientalis* exhibit ecological potential for colonization by *Asaia*. Presently, these two microbial species stand as promising candidates for the development of para-transgenic mosquitoes aimed at modifying the vector ability of mosquitoes. *Asaia* has the capacity for horizontal transmission through feeding on both larval and adult stages of mosquitoes, as well as vertical transmission through inheritance, making it a plausible candidate for further study [43,44]. Paratransgenic research centered on these two microorganisms holds the potential to significantly contribute to the advancement of mosquito control techniques. 

Taken together, this study revealed variations in the microbial community composition depending on the mosquito habitat, with the identification of species-specific biomarkers for each site. Spatiotemporal investigations into the mosquito microbiome offer potential applications in vector mosquito surveillance through the utilization of species biomarkers. Moreover, based on the findings of this study, it is recommended that Cx be included. *orientalis* in future Japanese encephalitis surveillance.

## Figures and Tables

**Table 1:** Mosquito collection.

Site Number	No. of Mosquito	
	Ct *	Co *
2	1	2
4	10	18
5	2	3
6	1	2
7	4	14
8	367	3
9	82	8
10	8	3
11	142	1
Total	617	54

2: 73-3, Sinjeop 2-gil, Bungnae-myeon, Yeoju-si, Gyeonggi-do, Republic of Korea; 4: 32 Sinjeong-ro 81beon-gil, Heungdeok-gu, Cheongju-si, Chungcheongbuk-do, 28292, Republic of Korea; 5: 419-6, Seongdeok-ri, Seongsan-myeon, Gunsan-si, Jeollabuk-do; 6: 29-5, Sinmae-gil, Hyoryeong-myeon, Gunwi-gun, Gyeongsangbuk-do; 7: 371-2, Pangok-ri, Sangju-si, Gyeongsangbuk-do; 8: 1240-2 Nakdongnam-ro, Saha-gu, Busan; 9: 36 Hakdong-gil, Suncheon-si, Jeollanam-do; 10: 79 Gangnam-ro, Jinju-si, Gyeongsangnam-do; 11: 5, Wonsan-ro 45beon-gil, Mokpo-si, Jeollanam-do; * *Ct: Culex tritaeniorhynchus*, *Co: Culex orientalis*.

**Table 2:** Species biomarkers of *Culex orientalis* and *Culex tritaeniorhynchus* by collecting sites Significant taxonomic was detected in 4 mosquito-collecting sites (Kruskal-Wallis H test, *p* < 0.05).

Collecting Site	Taxon Name	Taxon Rank	Taxonomy	*p*-Value	*p*-Value (FDR)	Relative Abundance
4	*Pantoea JTJJ_s group*	Species	Proteobacteria: Gammaproteobacteria: Enterobacterales: Erwiniaceae: Pantoea	0.03712	0.77587	38.69872
	*Pantoea_uc*	Species	Proteobacteria: Gammaproteobacteria: Enterobacterales: Erwiniaceae: Pantoea	0.0376	0.77587	9.08619
	*Erwinia persicina*	Species	Proteobacteria: Gammaproteobacteria: Enterobacterales: Erwiniaceae: Erwinia	0.02199	0.77587	8.98487
5	*Micrococcales*	Order	Actinobacteria: Actinobacteria_c	0.04942	0.7712	9.84041
	*Xanthomonadaceae*	Family	Proteobacteria: Gammaproteobacteria: Xanthomonadales	0.04942	0.7712	8.87366
	*Rhizobiaceae*	Family	Proteobacteria: Alphaproteobacteria: Rhizobiales	0.0376	0.7712	2.42987
6	*Gibbsiella quercinecans*	Species	Proteobacteria: Gammaproteobacteria: Enterobacterales: Enterobacteriaceae: Gibbsiella	0.03413	0.77587	29.75444
	*Serratia ficaria group*	Species	Proteobacteria: Gammaproteobacteria: Enterobacterales: Yersiniaceae: Serratia	0.03641	0.77587	27.36114
	*Enterococcaceae*	Family	Firmicutes: Bacilli: Lactobacillales	0.03712	0.77587	2.28524
10	*Entomospira*	Genus	Spirochaetes: Spirochaetia: Spirochaetales: Spirochaetaceae	0.03247	0.7706	59.82353
	*Sporomusa_uc*	Species	Firmicutes: Negativicutes: Selenomonadales: Sporomusaceae: Sporomusa	0.00169	0.7706	4.22798
	*EU939444_s*	Species	Firmicutes: Clostridia: Clostridiales: Lachnospiraceae: PAC001043_g	0.03385	0.7706	1.4542

## Data Availability

The metagenome reads obtained from this study were deposited in NCBI under BioProject PRJANA847343. All relevant data in this manuscript will be available from a public repository in NCBI.

## Supplementary Materials

The following supporting information can be downloaded at: https://www.mdpi.com/article/10.3390/insects15030201/s1, Figure S1: Alpha rarefaction graph; Table S1: Primer for amplifying V3–V4 region.
